# Cerebral venous sinus thrombosis as a complication of primary varicella infection in a child, case report

**DOI:** 10.1016/j.amsu.2021.103165

**Published:** 2021-12-11

**Authors:** Maysaa Badour, Eman Shhada, Ali Hammed, Sameer Baqla

**Affiliations:** a Pediatric University Hospital, Division of Neurology, Damascus, Syria; b Tishreen University Hospital, Department of Neurosurgery, Lattakia, Syria

**Keywords:** Chickenpox, Cerebral venous thrombosis, Child

## Abstract

**Introduction and importance:**

Chickenpox (Varicella) is a benign illness caused by varicella-zoster virus, predominant in childhood.

Chicken pox related neurological complications are seen in less than 1% cases of chickenpox.

Cerebral Venous thrombosis due to primary (VZV) infection is very rare, and it may occurs secondary to primary or re-activation the virus.

**Case presentation:**

We report a case of 5-year-old female complained of ataxia, vomiting, headache, and drowsiness 7 days after the onset varicella zoster infection. She had vesicular lesions with scab over the trunk and limbs.

**Clinical discussion:**

Neurological examination revealed left hemiparesis.

Her blood counts and metabolic parameters were normal.

Computed tomography brain showed hemorrhagic infarct in the left temporo-parietal region. Coagulation profile was normal. Magnetic resonance imaging (MRI) brain revealed hemorrhagic infarct in the same region. Magnetic resonance Venogram showed thrombosis of left transverse sinus and sigmoid sinus and internal jugular vein.

VZV- IgG antibody was positive but CSF VZV PCR (Polymerase chain reaction) was found to be negative.

Intravenous acyclovir for 15 days, and low-molecular-weight heparin for 3 days overlapped with oral Warfarin for 3months,. After 3 months follow up, the patient experienced a complete recovery. MRI repeated after 3 months showed recanalization of the sinuses.

**Conclusion:**

The pathogenic link of occurrence of CSVT after VZV infection is unclear, but some articles showed that it is related to direct endothelial damage by the virus.

Early recognition of this complication of VZV infection and prompt treatment is essential to prevent catastrophic complications.

## Highlights

• Chickenpox (Varicella) is a benign illness caused by varicella-zoster virus, predominant in childhood.
• Cerebral Venous thrombosis due to primary (VZV) infection is very rare.
• The pathogenic link of occurrence of CSVT after VZV infection is unclear.

## 1 Introduction

Chickenpox (Varicella) is a benign illness caused by varicella-zoster virus, predominant in childhood.

Chicken pox related neurological complications are seen in less than 1% cases of chickenpox [1]. The illness presents with fever and characteristic exanthematous vesicular skin rash [2]. Though it is a self limiting disease, occasionally serious complications can occur.

Varicella, like other infections, is associated with coagulopathy and venous thrombosis that lead to neurological complications [3]. Varicella related neurological complications are seen in less than 1% of cases.

Neurological complications frequently encountered are cerebellar ataxia and encephalitis. Less frequent complications are Guillian-Barré syndrome, meningoencephalitis, transverse myelitis, aseptic meningitis, ventriculitis, optic neuritis, post-hepatic neuralgia, herpes zoster ophthalmicus and peripheral motor neuropathy [4].

Cerebral Venous thrombosis due to primary (VZV) infection is very rare [3], and it may occurs secondary to primary or re-activation the virus.

Few cases have been reported in adults [[5], [6], [7], [8]] and only two cases in children [9,10] and hence this report.

This work has been reported in line with the SCARE criteria [11].

## 2 Case presentation

A previously healthy, 5-year-old female admitted to our neurology department 7 days after the onset varicella zoster infection, because of ataxia, vomiting, headache, and progressive drowsiness.

Neurological examination revealed left hemiparesis.

At the time of admission, she was drowsy, afebrile, and had vesicular lesions with scab over the trunk and limbs. Vital signs were normal. Child body weight 17 kg and height 115 cm.The laboratory investigations revealed complete blood count, liver function, hepatitis B and C serology, and CSF analysis within normal limits. Laboratory tests for collagen vascular disease and vasculitis, including antinuclear (ANA), antineutrophil cytoplasm (ANCA), and antiglomerular basement membrane antibodies, were also negative.

**Fig. 1:**
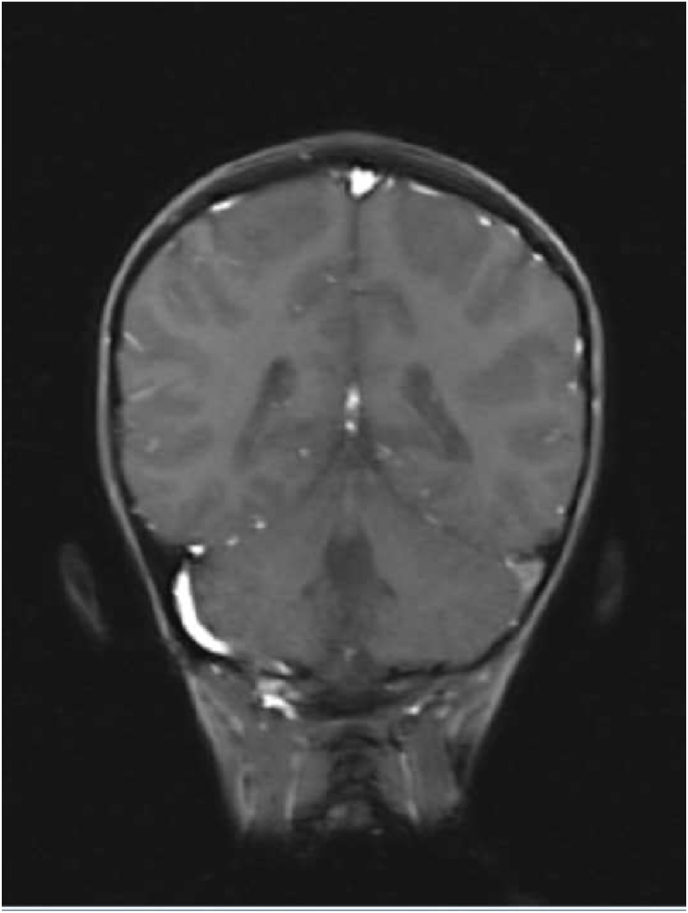
Coronal T1-weighted post-contrast images confirm filing defects within left transverse sinus and internal jugular vein.

Computed tomography brain showed hemorrhagic infarct in the left temporo-parietal region. Coagulation profile was normal. Magnetic resonance imaging (MRI) brain revealed hemorrhagic infarct in the same region. Coronal T1-weighted post-contrast images confirm filing defects within left transverse sinus and internal jugular vein (Fig. 1).

**Fig. 2:**
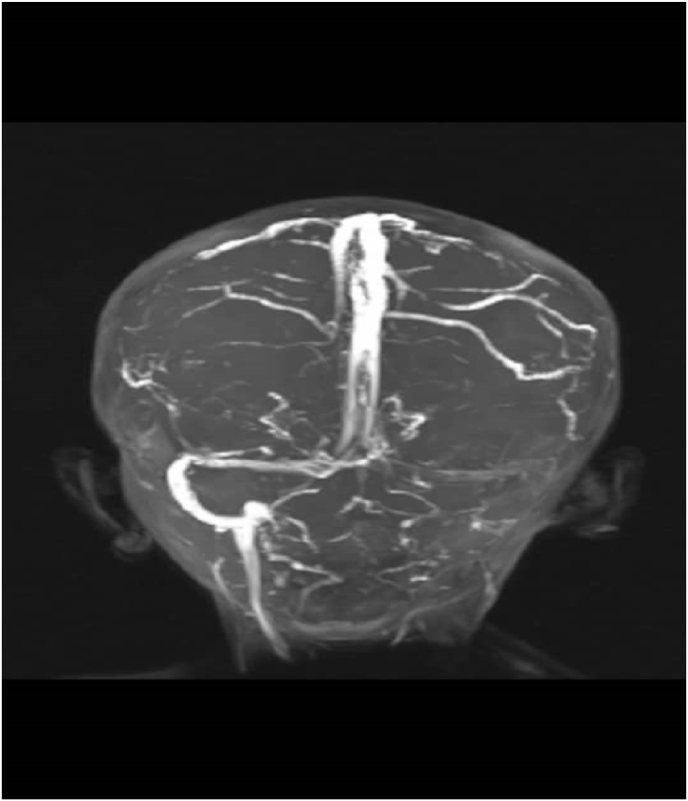
Magnetic resonance Venogram showed thrombosis of left transverse sinus and sigmoid sinus and internal jugular vein.

Magnetic resonance Venogram showed thrombosis of left transverse sinus and sigmoid sinus and internal jugular vein (Fig. 2).

Coagulation profile was performed including protein S and C, anti-thrombin II, prothrombin, and factor V leiden, which were negative, but the patient was carrier for MTHFR(C677T) mutation.

Although CSF VZV- IgG antibody was positive, CSF VZV PCR (Polymerase chain reaction) was found to be negative.

In view of straight forward clinical history of chicken pox rash followed by development of CVST along with virological evidence of VZV in CSF, the etiology seems to be viral (VZV) only.

After obtaining the patient's informed consent, treatment was planned.

Hence CSVT secondary to primary VZV was diagnosed intravenous acyclovir for 15 days, and low-molecular-weight heparin for 3 days overlapped with oral Warfarin for 3months. After 3 months follow up, the patient experienced a complete recovery. Follow-up MRI revealed recanalization of the sinuses after 3 months.

## 3 Discussion

VZV infection is a common febrile infection that occurs predominantly in childhood, the neurological complications are very rare [3] and were seen in less than 1% of all reported cases of VZV infection [12].

Neurological complications frequently encountered are cerebellar ataxia and encephalitis. Less frequent complications are Guillian-Barré syndrome, meningoencephalitis, transverse myelitis, aseptic meningitis, ventriculitis, optic neuritis, post-hepatic neuralgia, herpes zoster ophthalmicus and peripheral motor neuropathy [4].

The occurrence of cerebral sinus venous thrombosis CSVT secondary to primary VZV is also rare, though it can occur secondary to re-activation of VZV, and our patient was one of these rare cases with cerebral venous thrombosis [13], and she developed CSVT after 7 days of primary VZV. The pathogenic link of occurrence of CSVT after VZV infection is unclear, but some articles showed that it is related to direct endothelial damage by the virus [14,15].

The exact pathogenesis of varicella venous thrombosis is unknown. The same pathogenic mechanisms underlying arteriopathy may play a role in the development of cerebral sinus venous thrombosis. An acquired antibody-mediated hypercoagulable state resulting from decreased levels of natural anticoagulants such as protein S in the viremic phase of the infection is also postulated to provoke thrombosis [14,16]. Josephson et al. [17] in a cross-sectional study performed on 95 children showed that 43 children had antiphospholipid antibodies and some had a significant reduction in protein-S level post-varicella infection, describing it as transient varicella autoantibody syndrome.

Virological confirmation of VZV – associated cerebral vasculopathy includes CSF analysis for both VZV DNA and anti-VZV IgG antibody, although the diagnostic value of detecting anti-VZV IgG antibody in the CSF is greater than that of detecting VZV DNA [14]. Although a positive PCR for VZV DNA in CSF is helpful, a negative PCR does not exclude the diagnosis; only negative results in both VZV PCR and anti- VZV IgG antibody tests in the CSF can reliably exclude the diagnosis of VZV vasculopathy [18].

It is important to note that most of the infection-related CVSTs were found to be due to local infections such as ears, sinuses, mouth, face, or neck rather than systemic infection, which might support the argument for viral invasion as a possible cause of VrCVST [19].

Cerebral hernia caused by cerebral edema is the most common cause of death in patients with CVST. Although there were no deaths among the reported VrCVST patients, it is crucial to recognize malignant CVST when patients exhibit progressive symptoms, as these patients should be monitored closely and decompressive surgery should be promptly considered [20].

Patients with virus-associated vasculopathy are traditionally treated with intravenous acyclovir. The benefit of concurrent steroid therapy still awaits larger randomized controlled trials. Therefore, we did not administer steroids to our patient.

Our patient appeared with mild degree of fever associated with vesicles rash on her trunk and limbs which suggested VZV infection, 7 days after these symptoms, she developed neurological symptoms, so CSVT in our patient occurred during the primary infection and not as a delayed complication. After all investigations, we found sinus venous thrombosis confirmed the diagnosis. And the child has started the treatment with anti-viral and ant-coagulant. After 3 months of following up, the neurological symptoms were complete improved.

## 4 Conclusion

Primary VSV infection can be associated with vasculopathy and cerebral venous sinus thrombosis. Early recognition of this complication of VZV infection and prompt treatment are essential to prevent catastrophic complications.

## Ethical approval

This case report didn't require review by Ethics committee, pediatric university hospital, Damascus university, Faculty of medicine, Syria.

## Sources of funding

This research did not receive any specific grant from funding agencies in the public, commercial, or not-for-profit sectors.

## Author contribution

Maysaa Badour: Contribution to the paper: first author, data collection, writing the paper.

Eman Shhada: writing the paper.

Ali Hammed (corresponding author): Contribution to the paper: Writing the paper.

Sameer Baqla: Contribution to the paper:

Treatment and examination of the patient. Writing the paper.

## Research registration number

The case report at hand is not a first-in-man case report of a novel technology or surgical technique, therefore a registration of these case reports according to Declaration of Helsinki 2013 is not required.

## Guarantor

Ali Hammed.

## Consent for publication

Written informed consent was obtained from the patient's parents for publication of this case report and accompanying images. A copy of the written consent is available for review by the Editor-in-Chief of this journal.

## Provenance and peer review

Not commissioned, externally peer-reviewed.
